# Medical History from the Earliest Times

**Published:** 1895-06

**Authors:** 


### Reviews of Books.

*Medical History from the Earliest Times.* By EDWARD THEODORE WITHINGTON, M.B. Pp. viii., 424. London: THE SCIENTIFIC PRESS, LIMITED. 1894.

By writing a most interesting book, which treats of the history of medicine from the earliest times to the beginning of the present century, Dr. Withington has done well in creditably helping to rescue his country from its neglect of Medical History. The work displays not only great industry, but also considerable erudition, for the author has consulted, in all possible instances, his authorities and sources in the original; the bibliographical notes at the end of each chapter show how wide have been his reading and researches. Mediæval Medicine is treated with considerable fulness, but prehistoric and classical times are much more briefly considered. It is not stated in the preface that the chapters composing the work have appeared before; but we remember to have read the main portion of the text in the columns of *The Hospital* during the past year or two, and we are now glad to have them reprinted and elucidated with foot-notes. Having accomplished this much, we trust that Dr. Withington will be able to continue his labours, and in due time issue a larger work on the subject which may be fairly compared to those of Sprengel and Haeser.

